# P-1976. Incidence and Outcomes of Community-Onset Methicillin-Susceptible *Staphylococcus aureus* Bloodstream Infections in the Pre- and Post-COVID-19 Pandemic

**DOI:** 10.1093/ofid/ofae631.2134

**Published:** 2025-01-29

**Authors:** Jessica Chung, May Thet Hmu Tun, Khin Htet Htet Soe, Tin Mee Mee Aung, May Thu Zin, Franklin Liu, Monica Ghitan, Edward Chapnick, Yu Shia Lin

**Affiliations:** Maimonides Health, Brooklyn, New York; Maimonides Health, Brooklyn, New York; Maimonides Health, Brooklyn, New York; Maimonides Health, Brooklyn, New York; Maimonides Health, Brooklyn, New York; Maimonides Health, Brooklyn, New York; Maimonides Medical Center, Brooklyn, New York; Maimonides Medical Center, Brooklyn, New York; Maimonides Medical Center, Brooklyn, New York

## Abstract

**Background:**

Methicillin-susceptible *Staphylococcus aureus* (MSSA) is a pathogen linked to significant morbidity and mortality. CDC reported that the incidence of MSSA bloodstream infections (MSSA-BSI) significantly increased from 2012 to 2017, though mortality rates were unchanged. The COVID-19 pandemic may affect the incidence and mortality of MSSA-BSI due to the use of dexamethasone, delay in diagnosis due to quarantine, or the misdiagnosis of BSI as COVID-19. Our objective was to compare the incidence and outcomes of community-onset MSSA-BSI pre- and post-pandemic.

**Figure:**
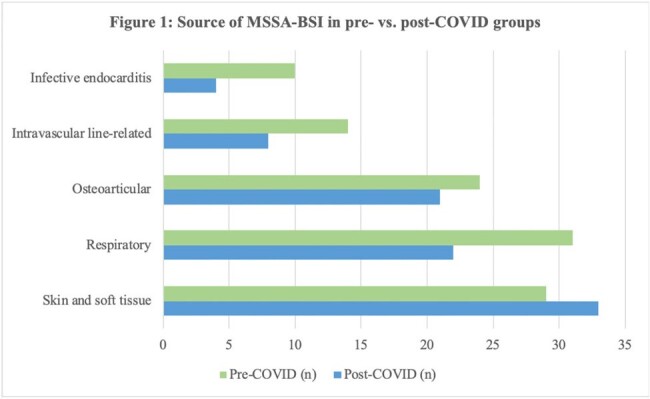

**Methods:**

This retrospective cohort study evaluated community-onset MSSA-BSI, defined as a positive blood culture within 72 hours of admission in hospitalized adults. The study population was divided into the pre-COVID group (June 1, 2018 to December 31, 2019) and the post-COVID group (January 1, 2022 to June 30, 2023). Patients with BSI other than MSSA, hospitalized within 30 days, transferred from another facility, and immunosuppressed were excluded. The Whitney Mann U test and chi-square test were used for the analysis.

**Figure:**
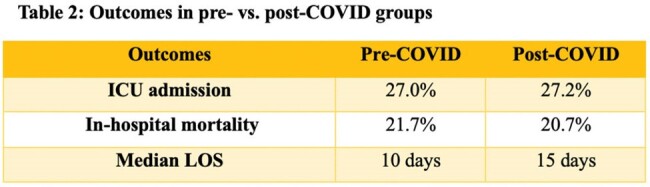

**Results:**

The study included 101 patients with MSSA-BSI: 48 in the pre-group, and 53 in the post-group. More than half of the patients were Caucasian males with a median age of 70 and 67 years in the pre- and post-group, respectively. The primary source of MSSA-BSI was skin and soft tissue, followed by respiratory, osteoarticular, and intravascular line-related infections.

The post-group had more cases who delayed seeking medical care and higher scores on the Charlson Comorbidity Index and Pitt bacteremia score. The post-group had a longer median length of stay (LOS) compared to the pre-group (15 vs. 10 days), p=0.022. The ICU admission rate and in-hospital mortality rate of pre- and post-group were 12.5% vs. 28% and 14.5% vs. 22.6%. However, neither of these measures were statistically different, p=0.051 and p=0.301 respectively.

**Conclusion:**

While the number of community-onset MSSA-BSI cases was similar, the post-group was more likely to delay seeking medical care and have longer LOS. The COVID-19 pandemic has impacted clinical care, leading to delayed medical attention, increasing disease severity. Our study highlights the importance of clinician awareness and patient education on early recognition of MSSA-BSI.

**Disclosures:**

All Authors: No reported disclosures

